# Infection of equine monocyte-derived macrophages with an attenuated equine infectious anemia virus (EIAV) strain induces a strong resistance to the infection by a virulent EIAV strain

**DOI:** 10.1186/s13567-014-0082-y

**Published:** 2014-08-09

**Authors:** Jian Ma, Shan-Shan Wang, Yue-Zhi Lin, Hai-Fang Liu, Qiang Liu, Hua-Mian Wei, Xue-Feng Wang, Yu-Hong Wang, Cheng Du, Xian-Gang Kong, Jian-Hua Zhou, Xiaojun Wang

**Affiliations:** State Key Laboratory of Veterinary Biotechnology, Harbin Veterinary Research Institute, Chinese Academy of Agricultural Sciences, Harbin, Heilongjiang 150001 China; Geriatrics Ward, First Hospital of Harbin Medical University, No23 Youzheng Street, Harbin, Heilongjiang 150001 China

## Abstract

The Chinese attenuated equine infectious anemia virus (EIAV) vaccine has successfully protected millions of equine animals from EIA disease in China. Given that the induction of immune protection results from the interactions between viruses and hosts, a better understanding of the characteristics of vaccine strain infection and host responses would be useful for elucidating the mechanism of the induction of immune protection by the Chinese attenuated EIAV strain. In this study, we demonstrate in equine monocyte-derived macrophages (eMDM) that EIAV_FDDV13_, a Chinese attenuated EIAV strain, induced a strong resistance to subsequent infection by a pathogenic strain, EIAV_UK3_. Further experiments indicate that the expression of the soluble EIAV receptor sELR1, Toll-like receptor 3 (TLR3) and interferon β (IFNβ) was up-regulated in eMDM infected with EIAV_FDDV13_ compared with eMDM infected with EIAV_UK3_. Stimulating eMDM with poly I:C resulted in similar resistance to EIAV infection as induced by EIAV_FDDV13_ and was correlated with enhanced TLR3, sELR1 and IFNβ expression. The knock down of TLR3 mRNA significantly impaired poly I:C-stimulated resistance to EIAV, greatly reducing the expression of sELR1 and IFNβ and lowered the level of infection resistance induced by EIAV_FDDV13_. These results indicate that the induction of restraining infection by EIAV_FDDV13_ in macrophages is partially mediated through the up-regulated expression of the soluble viral receptor and IFNβ, and that the TLR3 pathway activation plays an important role in the development of an EIAV-resistant intracellular environment.

**Electronic supplementary material:**

The online version of this article (doi:10.1186/s13567-014-0082-y) contains supplementary material, which is available to authorized users.

## Introduction

Equine infectious anemia virus (EIAV) is an equine lentivirus with a tropism primary for monocyte/macrophage lineage in vivo [1,2]. The clinical manifestation of equine infectious anemia (EIA), which is caused by EIAV infection, can be divided into an acute phase, a chronic phase, and an asymptomatic phase. The acute and chronic phases exhibit typical viremia accompanied by a high fever, anemia, thrombocytopenia, edema, and weight loss. The infected equines usually enter the life-long asymptomatic carrier state after 8–12 months. However, the virus maintains a low level of stable replication in tissues that are enriched with monocytes [1,3,4]. Stress or immunosuppression can increase the EIAV replication level in asymptomatic carriers and thus lead to a recurrence of EIA. Because most EIAV-infected equines become asymptomatic carriers due to immune control of the infection, EIAV has become a unique lentivirus model for studies investigating the immune control of lentivirus infection and its pathogenesis. In addition, most asymptomatic EIAV-infected horses demonstrate a significant resistance to infections caused by different pathogenic EIAV strains [1,5]. This finding suggests that the EIAV system can provide a model for studying key immune factors that are involved in resistance to lentiviral infection, which can be used for the research and development of preventive lentivirus vaccines.

EIAV_FDDV13_ is an attenuated EIAV vaccine strain that induces immune protection in approximately 80% of vaccinated animals in laboratory and clinical studies [6,7]. An understanding of the mechanism that underlies the induction of immune protection imparted by this attenuated vaccine strain will be useful for elucidating the immune protective mechanism that is responsible for lentivirus infection. Furthermore, the induction of immune protection results from the interaction between viruses and hosts. Therefore, the cellular responses of equine macrophages, the primary target cells of EIAV in vivo, should be evaluated after being infected by EIAV.

In this study, we examined the infection characteristics of a pathogenic EIAV strain EIAV_UK3_ on equine macrophages pre-infected by EIAV_FDDV3_ in vitro. We confirmed that EIAV_FDDV13_ induced a strong resistance to the subsequent EIAV_UK3_ infection in equine macrophages. Noticeably, in addition to the previously reported mechanism, i.e. masking viral receptor ELR1 by the SU protein of EIAV [8,9], our results revealed that up-regulation of the soluble EIAV receptor and interferonβ (IFNβ) by activated Toll-like receptor 3 (TLR3) are also largely involved in the resistance to EIAV_UK3_ infection induced by EIAV_FDDV13_.

## Materials and methods

### Cells and viral strains

eMDM and fetal donkey dermal (FDD) cells were used in this study as target cells for EIAV. The eMDM were prepared from the PBMC of one donor horse. The red blood cells (RBC) of equine animals have a faster sedimentation velocity than the white blood cells (WBC) in natural sedimentation in heparinized whole blood without any other treatment. After at least 30 min natural sedimentation, RBC will stay in the bottom of the flask, but most WBC including monocytes will stay on the upper layer with the plasma. Thus, the upper plasma layer supernatant including WBC was obtained from freshly collected, heparinized whole horse blood following natural sedimentation at room temperature for 30 min. The blood cells in the supernatant were isolated with centrifugation at 1000 rpm. After 2–3 washes with cold phosphate-buffered saline (PBS), the cells were incubated in RPMI 1640 culture medium (Gibco: Invitrogen Corporation, Carlsbad, CA, USA) supplemented with 10% horse serum (Hyclone, Logan, USA) and 40% fetal bovine serum (FBS) (Hyclone) at 37 °C and 5% CO_2_. After 24 h of incubation, non-adherent and loosely adherent cells were removed by washing with cold PBS quickly for three times. The remaining adherent cells were detached with normal saline at 37 °C and seeded into 96-, 24-, or 6-well microplates (Costar, Corning, USA) for 24 incubation at 1 × 10^5^, 1 × 10^6^, and 5 × 10^6^ cells/well, respectively, depending on the experiment. After 48 h incubation, most adherent cells had differentiated into macrophages (see Additional file 1 to identify the differentiation from monocytes to macrophages by specific immune-staining) and were further used for EIAV infection assays. The FDD cell cultures were prepared and stored in our laboratory. FDD cells were prepared from EIAV negative fetal donkeys and cultured in minimal essential medium (α-MEM, Gibco) containing 2 mM L-glutamine, 10% heat-inactivated FBS, 100 IU penicillin, and 100 μg/mL streptomycin (Gibco) as previously described [10].

Two EIAV strains were used in this study. EIAV_FDDV13_, an attenuated vaccine strain of EIAV, was developed by passaging EIAV_DLV121_, a Chinese donkey leukocyte-adapted attenuated strain of EIAV, in FDD cells for 13 generations. A protective test demonstrated that EIAV_FDDV13_ induced protection from disease in approximately 80% of vaccinated horses [7], and the strain remained stably attenuated in the hosts [11]. EIAV_UK3_, a pathogenic strain of EIAV, is an infectious clone constructed from the backbone of an EIAV_wyoming_ strain [12]. This infectious clone was kindly provided by Dr R. Montelaro of the Center for Vaccine Research at the University of Pittsburgh. The genomic variation at the nucleotide level between EIAV_FDDV13_ and EIAV_UK3_ was approximately 25%.

### Quantification of EIAV load and detection of viral replication

Real-time quantitative reverse transcription PCR (RT-PCR) and reverse transcriptase activity (RT) assays were used to identify the EIAV load. The relative titers of EIAV_FDDV13_ and EIAV_UK3_ were comparable and consistent when measured with these methods. Therefore, a quantitative RT-PCR assay of viral genomic RNA was used to quantify the loads of EIAV_FDDV13_ and EIAV_UK3_ according to previously described procedures [6,13].

The infectious titer of the two EIAV strains was tested using the median tissue culture infective dose method (TCID_50_). Fifty microliters of viral supernatants that were serially diluted by 10^−4^, 10^−5^, 10^−6^, and 10^−7^ was added to eight wells of 96-well flat-bottom plates; each well contained 5 × 10^5^ FDD cells. After an initial incubation of 2 h, viruses in the culture medium were removed with three washes with serum-free medium. Fresh cell culture medium was then added to the cultures, and the infected cells were incubated continuously. Four days later, EIAV growth was monitored by measuring viral RT activity using a Roche RT detection kit (Roche, Basel, Switzerland). Optical density values two-fold higher than those determined for the negative control were considered to indicate viral replication. The TCID_50_ value of the virus was determined as described by Reed and Muench [14].

To examine the proliferation profiles of EIAV in eMDM, 1 × 10^5^ cells were infected with 1 × 10^3^ TCID_50_ of EIAV_FDDV13_ (amounted to approximately 1 × 10^7^ viral RNA copies of EIAV_FDDV13_) or 1 × 10^3^ TCID_50_ of EIAV_UK3_ in a 96-well microplate as indicated in the text. The culture medium was exchanged for fresh culture medium after 2 h of infection. The cells in some wells were used to determine the intracellular viral RNA copies at 3 h after infection by EIAV. These cells were washed three times with PBS and treated with trypsin-EDTA (0.25% trypsin, 5 mM EDTA) at room temperature for 5 min to remove adherent virus that had not entered the cells. The other cells that were used to examine the proliferation profiles were further incubated for 2, 3, 4, 5, 6, or 7 days, after which the cell culture supernatants were collected. Triplicate wells were used for each detection time point. Total RNA was extracted from the harvested cells and culture supernatants using Trizol (Invitrogen, Carlsbad, CA, USA) or the QIAamp Viral RNA Mini Kit (Qiagen, Hilden, Germany) and was processed for cDNA synthesis using M-MLV reverse transcript kit (Invitrogen) using 100 ng of RNA template. The cDNA obtained was used for qPCR analysis. The replication kinetics of the viruses was determined in three independent experiments.

### Co-infection measured by RNA HIS

EIAV positive-strand RNA in infected cells was detected with a QuantiGene ViewRNA Plate-based Assay Kit (Panomics, Silicon Valley, USA). Two sets of specific probes that targeted the EIAV genome at nucleotides 2065 to 3210 of EIAV_FDDV13_ (GenBank accession # GS00329) and nucleotides 1,210 to 2356 of EIAV_UK3_ (GenBank accession # AF016316) were designed and provided by Panomics. The divergences between the two targeted regions of these two EIAV strains (EIAV_FDDV13_ and EIAV_UK3_) are 23.1% and 27.6%, respectively. The eMDM were plated in 96-well plates (Costar) and simultaneously infected with these two EIAV strains. At 48 h after initial EIAV infection, the cells were fixed with 4% formaldehyde and dehydrated in ethanol. During the detection process, the cells were rehydrated, permeabilized, digested with protease and hybridized with the specific probes as recommended by the manufacturer. Confocal microscopy and image acquisition were performed with a Leica TCS SP5 (Leica, Wetzlar, Germany).

### Measurement of mRNA expression by the branched DNA technique and real-time quantitative RT-PCR

The branched DNA (bDNA) technique was used to measure the expression levels of multiple genes in the cultured cells. The specific oligonucleotide probe sets for the target genes included equine TLR3, TLR7, TLR8, TLR9, IFNα1, IFNβ, ELR1, and β-actin, which were used with the QuantiGene 2.0 Reagent Systems designed and provided by the manufacturer (Panomics). Information regarding the probe sets is provided in Additional file 2. The amounts of multiple target mRNA in each sample were simultaneously determined by measuring the wavelengths of color-coded microspheres and the intensities of the luminescent emission of streptavidin-conjugated R-phycoerythrin using a Luminex 200 (Molecular Devices, Silicon Valley, USA). All data obtained from the Luminex 200 were analyzed using the Luminex IS2.3 program. A total of 100 events per region were collected. For all of the samples analyzed with the bDNA assay, background signals determined in the absence of target mRNA were subtracted from the signals obtained in the presence of target mRNA. The expression levels of the intracellular mRNA were normalized to β-actin. Changes in gene expression were calculated by the following method: fold changing value = (copies of target mRNA/copies of β-actin mRNA)_treated sample_/(copies of target mRNA/copies of β-actin mRNA)_untreated control_, and were presented as the log_2_ mean fold changing value in the results. The ratio of copies between target mRNA and β-actin mRNA for the untreated control was used as the calibrator and assigned a fold-change expression value of 1. Three independent experiments were performed for each treatment. In addition, the gene expression of the “house-keeping” β-actin gene used in this study was detected and compared among different eMDM: those infected with EIAV_FDDV13_ and EIAV_UK3_ or treated with Poly I:C. The eMDM under different treatments were harvested at different internal times and counted. eMDM with the same numbers were used to quantify mRNA copies of β-actin by Quantitative real-time RT-PCR.

Quantitative real-time RT-PCR using a kit Platinum® SYBR® Green qPCR SuperMix-UDG (Invitrogen) which utilizes SYBR Green as a detector was performed by using an MxPro 3005p qPCR system (Stratagene, La Jolla, CA, USA) to analyze the expression of ELR-IN, an alternative splicing isoform of the EIAV receptor ELR1 [15], TLR3, and β-actin. Primers were designed based on the nucleotide sequence of ELR-IN (GenBank accession # EF190264) to specifically distinguish ELR-IN from ELR1. The sequences of the primers were IN-FW, 5′ GGAGAGTCCTTCAGACCTGAGTTCAC3′; IN-RV, 5′ CGCTGCACCTAGGAGAGAAGATTGGC3′. The primers for TLR3 mRNA (GenBank accession # NM_001081798.1) were TLR3-FW, 5′ GGGCAAGAACTCACAGGTCAG 3′; TLR3-RV, 5′ CAAACCAGGCAATGCTTTCAC 3′. The primers for β-actin mRNA were F2, 5′ CGACATCCGTAAGGACCTGTA 3′; R2, 5′ CATCTGCTGGAAGGTGGACAA 3′. Total RNA was extracted from the harvested cells using Trizol (Invitrogen) and was processed for cDNA synthesis using an M-MLV reverse transcript kit (Invitrogen,) using 100 ng of RNA template. The cDNA obtained was used for qPCR analysis. qPCR was conducted under the following conditions suggested by the manufacturer of Platinum SYBR Green qPCR SuperMix-UDG kit (Invitrogen): initial preincubation at 50 °C for 20 s; 95 °C for 10 min; 40 cycles of 95 °C for 30 s and 60 °C for 1 min; and one cycle of 95 °C for 1 min, 55 °C for 30 s and 95 °C for 30 s for signal sampling. Linear regression analysis of the standard curve and the β-actin values was used to estimate the ELR-IN and TLR3 mRNA level in the samples.

### Enzyme-linked immunosorbent assay for equine IFN-α/β

Enzyme-linked immunosorbent assays (ELISA) for the analysis of equine IFNα and IFNβ proteins were performed as described in the protocol provided by the manufacturer (Uscn Life Science, Wuhan, China). The plate was read with a microplate reader VERSAmax (Molecular Devices). The protein expression levels of IFN were measured by ELISA as pg/mL calibrated with a set of standards provided by this kit, and the changes in protein expression levels in each sample were calculated using the following formula: fold changing value = the amount of target protein in the treated samples/the amount of target protein in untreated samples, and the mean values of fold change were converted to log2. The amount of IFN proteins in the untreated control was used as the calibrator and assigned a fold-change expression value of 1. All reactions were performed in triplicate.

### EIAV infection of poly I:C-treated eMDM

eMDM were cultivated in 96-well plates and treated with either 0.5 μg/mL poly I:C (Sigma-Aldrich, St Louis, USA) or the same volume of PBS as a control. The culture supernatant was removed 12 h after the treatment, and fresh medium was added. The cells were then infected with 1 × 10^3^ TCID_50_ of EIAV_UK3_. After 2 h of incubation, the culture medium was removed, and the cells were washed three times with PBS before incubation in fresh medium for an additional 72 h. The viral copy numbers in the culture medium of triplicate wells were quantitatively analyzed by qPCR.

### RNA interference of TLR3 expression

To knock down TLR3 expression, a TLR3-specific small interfering RNA (siRNA-TLR3) and a control siRNA (siRNA-C) with scrambled sequences were synthesized by RiboBio, Guangzhou, China. The target sequence was 5′-GGACCTTGGCCTTAATGAA-3′, and the product number for siRNA-C was Ncontrol_05815. Primary eMDM were plated in 96-well plates at 1 × 10^5^/well and transfected with either 50 nM siRNA-TLR3 or 50 nM siRNA-C using the transfection reagent FuGENE HD (Promega, Madison, USA) according to the manufacturer’s protocol. The cells were harvested at 48 h after transfection and evaluated for the efficacy of TLR3 mRNA knockdown.

### Statistical analysis

All experiments in the present study were replicated at least three times unless specifically indicated. The results in the figures are presented as the mean ± SEM. Significant differences between samples or groups were determined with the student’s *t*-test. The statistical analysis was performed using SAS 8.1 software.

## Results

### EIAV_FDDV13_ induced strong resistance to the infection of EIAV_UK3_ on eMDM

**Figure 1:**
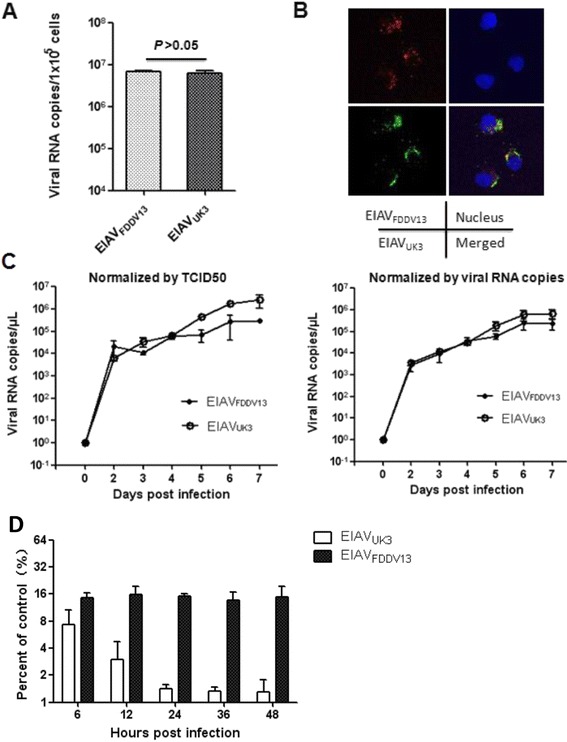
**EIAV**_**FDDV13**_ **induced strong resistance to subsequent infection of EIAV**_**UK3**_ **in eMDM. (A)** Comparison of intracellular viral levels in the early phase of infection. The same infectious dose (1 × 10^3^ TCID_50_/well) of the attenuated EIAV strain EIAV_FDDV13_ and the pathogenic strain EIAV_UK3_ was used to infect eMDM in 96-well microplates. At 3 hpi, the copy numbers of intracellular EIAV RNA were measured with qPCR. **(B)** Co-infection of eMDM with EIAV_FDDV13_ and EIAV_UK3_. Cultivated eMDM were simultaneously infected with equivalent TCID_50_ of EIAV_FDDV13_ and EIAV_UK3_. Intracellular viruses were detected with ViewRNA in situ hybridization at 48 hpi and are indicated as fluorescently labeled granules (red for EIAV_FDDV13_ and green for EIAV_UK3_). **(C)** Comparison of the replication kinetics of EIAV_FDDV13_ and EIAV_UK3_ in eMDM. Stocks of these two viruses with an equal TCID_50_ or equal RNA copy numbers were used to infect eMDM as indicated. Viruses in the culture medium were quantified as viral RNA copy numbers at various time points up to 7 dpi. **(D)** Quantitative analysis of the restriction of EIAV_UK3_ or EIAV_FDDV13_ infection by prior EIAV_FDDV13_ or EIAV_UK3_ infection. Cultivated eMDM were pre-infected by 1 × 10^3^ TCID_50_ of EIAV_FDDV13_ or EIAV_UK3_ in 96-well plates. The culture medium was changed after 2 h of infection. The cells were washed three times with PBS at 6, 12, 24, 36 and 48 hpi and were infected with equivalent infectious amounts of EIAV_UK3_ or EIAV_FDDV13_ diluted in the culture medium. After another 48 h, the culture supernatant was collected and the viral loads of the subsequently infected virus in the medium were measured as the copy numbers of EIAV RNA. The Y-axis of the graph represents the ratio of viral RNA copies to the number of viral RNA copies in cells without pre-infection with EIAV.

To examine the ability of EIAV_FDDV13_ to interfere with the infection of EIAV_UK3_, a pathogenic EIAV strain, EIAV_FDDV13_ and EIAV_UK3_ viruses at the same infectious titer were first used to infect equal numbers of eMDM The infection and replication patterns of the two strains were analyzed with qRT-PCR. The intracellular viral copy numbers determined in early-phase infection (3 hours post-infection (hpi)) indicate that the number of viruses that entered the cells was similar for the two strains (Figure 1A). The co-presence of both EIAV_FDDV13_ and EIAV_UK3_ in infected cells was examined by ViewRNA in situ hybridization using probes labeled with different fluorescent dyes. The images of EIAV in infected cells presented in Figure 1B clearly demonstrate that these two viral strains co-infected and replicated in common macrophages. Furthermore, the replication kinetics of EIAV_FDDV13_ and EIAV_UK3_ in the cell culture medium during the seven-day post-infection period was examined using inocula normalized by either TCID_50_ or RNA copy numbers. Besides a slight decreased viral load of EIAV_FDDV13_ on 4 days post infection (dpi), the two EIAV strains grew equally well in cultivated eMDM with similar replication kinetics regardless whether initially normalized by infectivity or viral particles (Figure 1C). In addition, the difference in the ratio of RNA copy number/infectious titer in the viral stocks, which represents the difference in infectivity of EIAV in the target cells [16], was measured. The measured ratios for EIAV_FDDV13_ and EIAV_UK3_ were 33 133.73 ± 2204.662 and 29 245.28 ± 2037.972, respectively, with no significant difference (*P* = 0.14). These results indicate that the two EIAV strains used in this study replicated in eMDM with similar kinetics and similar cell-cell spreading efficacies.

Afterwards, the restriction of a subsequent infection with EIAV_UK3_ by pre-infection with EIAV_FDDV13_ or a subsequent infection with EIAV_FDDV13_ by pre-infection with EIAV_UK3_ was investigated. The viral RNA copy numbers of EIAV_UK3_ or EIAV_FDDV13_ in eMDM pre-infected with EIAV_FDDV13_ for 6, 12, 24, 36 and 48 h were measured and compared with the EIAV RNA copy numbers in control groups (cells not treated with pre-infection of EIAV). As shown in Figure 1D, the RNA copy numbers of EIAV_UK3_ were markedly reduced by 92.68 ± 3.35% by prior infection with EIAV_FDDV13_ for 6 h compared with the control group. The restriction effect increased by approximately 5-fold with prolonged pre-infection time until 24 h, at which point the reduction in RNA copy number was 98.71 ± 0.48%. In contrast, the resistance induced by EIAV_UK3_ to subsequent infection by EIAV_FDDV13_ was much weaker. The restriction induced by EIAV_UK3_ was approximately 85% throughout the detection period and was 10-20-fold lower than that induced by EIAV_FDDV13_ after 24 h of pre-infection. Meanwhile, there was no significant difference in apoptosis of eMDM induced by the infection of these two EIAV strains (see Additional file 3), which ruled out the possible effect of cell degradation on the aforementioned difference in viral RNA replication. These results demonstrate that the initial infection of EIAV_FDDV13_ in eMDM induced a strong resistance to the subsequent infection of EIAV_UK3_.

### Infection of eMDM with EIAV_FDDV13_ and EIAV_UK3_ differentially influenced the expression of ELR1 and soluble ELR1 (sELR1)

**Figure 2:**
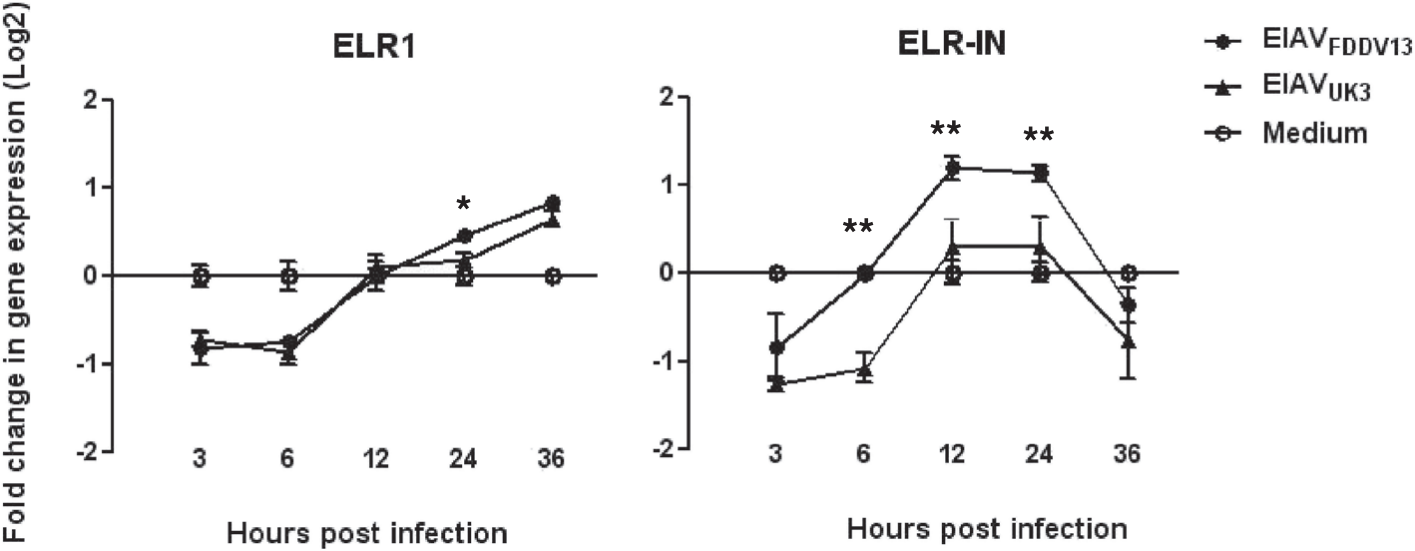
**Regulation of membrane-bound and soluble EIAV receptor expression in eMDM by EIAV**_**FDDV13**_ **and EIAV**_**UK3.**_ **(A)** Regulation of membrane-bound EIAV receptor ELR1 expression. Total RNA was extracted from eMDM that were infected with equal infectious titers of either EIAV_FDDV13_ or EIAV_UK3_ for various times. ELR1 mRNA was quantified with a branched DNA (bDNA) assay. **(B)** Regulation of soluble EIAV receptor expression. ELR-IN is a transcriptional variant of ELR1 that encodes a soluble form of the receptor (sELR1). The expression levels of ELR-IN in eMDM infected with either EIAV_FDDV13_ or EIAV_UK3_ for various times were quantified with real-time qPCR. The values of Y axis were treated by Log2. *P < 0.05, **P < 0.01.

The EIAV receptor ELR1 has been shown to play an important role in induction of superinfection resistance (SIR) [9]. To investigate the role of ELR1 in the infection resistance induced in eMDM by EIAV_FDDV13_, a quantitative analysis of ELR1 mRNA levels in eMDM up to 36 hpi with EIAV_FDDV13_ was performed. As a provirus-derived pathogenic strain, the inductive activity of EIAV_UK3_ was also investigated to compare it with that of EIAV_FDDV13._ As shown in Figure 2A, the kinetics of ELR1 mRNA expression was similar in eMDM infected with the two viruses: ELR1 expression was first down-regulated and then up-regulated. However, both down-regulation and up-regulation occurred over a limited range, and down-regulation only occurred within 6 hpi, suggesting a limited involvement of ELR1 regulation in EIAV-induced infection resistance. Meanwhile, β-actin expression in eMDM was not influenced after being infected with EIAV_FDDV13_ and EIAV_UK3_ (see the result in Additional file 4). It ensured the validity of the data for gene expression dynamics obtained in this study.

We recently identified an alternative splicing variant of ELR1 transcripts that retained a fragment of intron 6. This sliced transcript, termed as ELR-IN (GenBank accession # EF190264), accounts for a large proportion of ELR1 transcript variants (approximately 50% of the ELR1 transcript) and creates an isoform with four different amino acid residues and then a premature stop codon 14 residues upstream of the predicted membrane spanning domain. The truncated ELR1 protein is predicted and confirmed as a soluble form of ELR1. A separate study revealed that sELR1 appeared to inhibit EIAV infection in cultivated host cells [15]. Therefore, the regulation of ELR-IN expression by EIAV infection was tested. The levels of sELR1 mRNA in eMDM infected with EIAV_FDDV13_ were significantly higher than in EIAV_UK3_-infected cells and uninfected control cells within 12–24 h after infection, differently than that was observed for transmembrane ELR1 (Figure 2B). This result suggests that the up-regulation of sELR1 expression in eMDM after infection with EIAV_FDDV13_ may contribute to induction of infection resistance by this EIAV strain.

### The expression levels of viral nucleic acid-recognizing TLR and type I interferons were differentially regulated by EIAV_FDDV13_ and EIAV_UK3_ infection in eMDM

**Figure 3:**
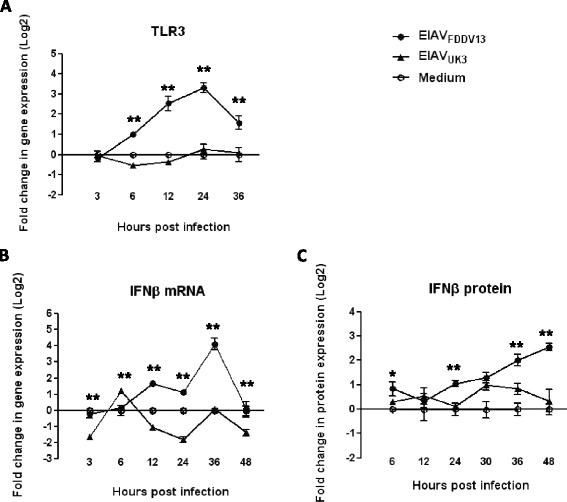
**Regulation of TLR3 and IFNβ expression in eMDM by EIAV**_**FDDV13**_ **and EIAV**_**UK3.**_ eMDM infected with equal infectious titers of either EIAV_FDDV13_ or EIAV_UK3_ for various times were selected. Some cells were treated with Trizol, and the total RNA was extracted to quantify TLR3 **(A)** and IFNβ **(B)** mRNA expression with the bDNA assay. The remaining cells were lysed with RIPA cell lysis buffer, and the protein levels of IFNβ in the eMDM lysates were measured with an ELISA kit **(C)**. The values of Y axis were treated by Log2. **P < 0.01.

Because macrophages, the primary target cells of EIAV in vivo, are important in immune responses and are essential for the effects of TLR activation on the innate anti-virus mechanisms of immunocytes, the activation of TLR3, TLR7, TLR8 and TLR9, which are activated by single-stranded or double-stranded foreign RNA or foreign DNA, was analyzed. As shown in Figure 3A, EIAV_FDDV13_ significantly up-regulated TLR3 mRNA expression, with a peak (8- to 10-fold) at 24 hpi. However, EIAV_UK3_ had no effect on TLR3 mRNA levels. With regards to TLR7 and TLR8 expression, EIAV_UK3_ up-regulated TLR expression approximately 0.5- to 1.5-fold at 12 to 24 hpi, but EIAV_FDDV13_ did not exhibit such an effect. No significant difference in TLR9 expression was observed in eMDM infected with EIAV_FDDV13_ and EIAV_UK3_; both strains moderately up-regulated TLR9 expression (see Additional file 5).

Studies have shown that type I interferons are associated with intracellular TLR activation and that they are part of the downstream products of TLR signaling pathways [17–19]. Given the obvious up-regulation of TLR3 mRNA expression by EIAV_FDDV13_ infection and the antiviral activity of type I interferons, changes in the expression of IFNβ and IFNα in eMDM after infection with EIAV_FDDV13_ and EIAV_UK3_ were evaluated. As shown in Figure 3B, there were significant differences in IFNβ expression in eMDM infected with the two strains. At 24 h after the infection of eMDM with EIAV_FDDV13_, IFNβ expression was up-regulated by 10- to 20-fold at the mRNA level and 4- to 6-fold at the protein level compared with the mock-treated control; the timing of this up-regulation was correlated with the temporal up-regulation of TLR3 expression in eMDM infected with EIAV_FDDV13_. In contrast, IFNβ mRNA and protein expression in eMDM infected with EIAV_UK3_ did not differ significantly from those in the mock-treated control. Although EIAV_UK3_ infection up-regulated IFNα expression (approximate 3 folds in protein level), infection with EIAV_FDDV13_ basically did not (Additional file 6). Based on the antiviral effect of IFNβ, it is likely that the up-regulation of IFNβ expression is positively correlated with the strong infection resistance induced by EIAV_FDDV13_.

### TLR3 activation in eMDM induced by poly I:C resulted in increased sELR1 and IFNβ mRNA expression

**Figure 4:**
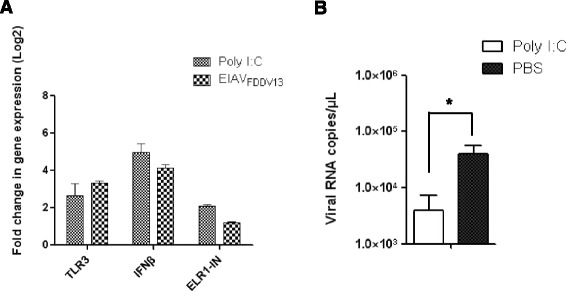
**Effects of poly I:C on TLR3, type I interferons and sELR1 expression and EIAV replication. (A)** Effects of poly I:C on TLR3, IFNβ and sELR1 expression. eMDM were treated with poly I:C (0.5 μg/mL) for 12 h. The expression levels of specific mRNA in the cells were analyzed using the bDNA assay. The values of Y axis were treated by Log2. **(B)** Effects of poly I:C on EIAV_UK3_ replication. eMDM were treated with either 0.5 μg/mL poly I:C or the same volume of PBS as a control for 12 h; the cells were then infected with EIAV_UK3_. Viral RNA copy numbers in the culture medium were measured with real-time qPCR at 72 hpi. *P < 0.05.

Considering the correlation between strong induction of infection resistance and elevated TLR3 and IFNβ expression induced by attenuated EIAV_FDDV13_ as well as the existence of a signaling pathway linking TLR3 activation with IFNβ expression, the effect of TLR3 activation on infection resistance induced by EIAV_FDDV13_ was mimicked by treating eMDM with poly I:C, a TLR3 ligand. Treatment with poly I:C at 0.5 μg/mL up-regulated TLR3 mRNA expression to similar levels as those observed in eMDM infected with EIAV_FDDV13_ (Figure 4A). In addition, replication of the pathogenic EIAV strain EIAV_UK3_ in poly I:C-treated cells declined by approximately 90% compared with untreated cells (Figure 4B). By the way, β-actin expression was at a similar level in eMDM after being treated with poly I:C or being infected with the two EIAV strains used in this study (see Additional file 4). This could also ensure the validity of the data for gene expression dynamics obtained in this experiment.

Intriguingly, following the specific activation of TLR3 by poly I:C in eMDM, the mRNA expression levels of both IFNβ and sELR1 were up-regulated. The up-regulation of IFNβ was similar to that observed in EIAV_FDDV13_-infected eMDM (Figure 4A). Moreover, expressions of IFNα and TLR7-9, which show lower intensity induced by attenuated EIAV_FDDV13_ than that induced by the virulent EIAV_UK3_ or show a limited difference between these two EIAV strains, were also not up-regulated after poly I:C treatment (data not shown). These results indicate that enhanced IFNβ and sELR1 expression can be induced by TLR3 activation, which in turn promotes resistance of the target cells to EIAV infection.

### TLR3 activation in eMDM played an important role in induction of infection resistance to EIAV_UK3_

**Figure 5:**
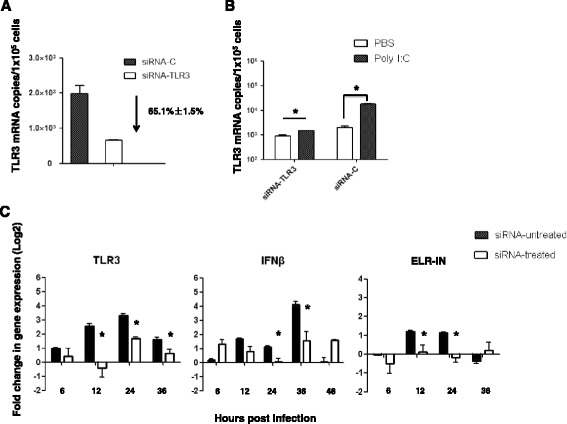
**Effects of TLR3 mRNA knockdown on sELR1 and INFβ expression in eMDM. (A)** Knockdown of TLR3 expression in eMDM with siRNA. At 48 h after transfection with siRNA targeting equine TLR3 (siRNA-TLR3), the expression level of TLR3 mRNA was quantified with real-time qPCR. siRNA with a scrambled sequence (siRNA-C) was used as the control. **(B)** The induction of TLR3 expression by poly I:C in siRNA-TLR3-transfected eMDM. The cells were transfected with siRNA-TLR3 or siRNA-C. TLR3 mRNA was measured with real-time qPCR 12 h after stimulation with 0.5 μg/mL poly I:C. **(C)** Kinetics of TLR3, sELR1 and INFβ mRNA expression induced by EIAV_FDDV13_ in eMDM transfected with siRNA-TLR3. Cells were transfected with siRNA-TLR3 or siRNA-C. After 48 h of incubation, EIAV_FDDV13_ was added to the cells, and total RNA was extracted at different times after infection. The mRNA copies were specifically amplified and quantified with bDNA (for TLR3 and INFβ) and qPCR (for sELR1). The values of Y axis were treated by Log2. *P < 0.05.

To confirm that up-regulated TLR3 expression plays a crucial role in the induction of infection resistance in EIAV_FDDV13_-infected eMDM, TLR3 expression in eMDM was knocked down with siRNA. As shown in Figure 5A, TLR3 transcription was reduced by approximately 65% in eMDM transfected with horse TLR3 siRNA (siRNA-TLR3) compared with cells transfected with scrambled RNA control siRNA-C. When the siRNA-TLR3-transfected cells were treated with 0.5 μg/mL poly I:C for 12 h, only a slight up-regulation of TLR3 mRNA expression (0.5-fold) was observed, whereas an approximately 8.0-fold increase in TLR3 mRNA expression was detected in siRNA-C-transfected cells (Figure 5B). These results indicate that TLR3 expression was substantially suppressed by its specific siRNA. The effect of EIAV_FDDV13_ on TLR3, sELR1 and IFNβ expression was evaluated after TLR3 knockdown. As shown in Figure 5C, compared with the effect of EIAV_FDDV13_ on eMDM that were not treated with siRNA, the up-regulation of these three factors was greatly diminished in cells transfected with specific siRNA-TLR3.

**Figure 6:**
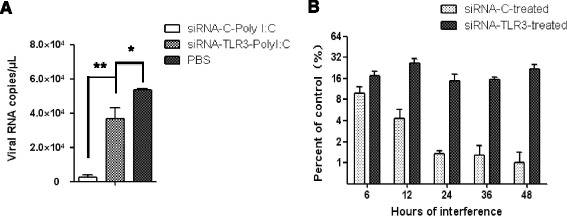
**EIAV replication and induction of infection resistance in eMDM after TLR3 mRNA knockdown. (A)** The inhibitory effect of poly I:C on EIAV_UK3_ replication in eMDM transfected with specific siRNA-TLR3 or control siRNA-C. After transfection with siRNA for 48 h, the cells were stimulated with 0.5 μg/mL poly I:C for 12 h before infection with EIAV_UK3_. Viral RNA copies were measured with qPCR at 72 hpi. **(B)** The effect of TLR3 mRNA knockdown on the resistance to subsequent viral infection induced by EIAV_FDDV13_. eMDM transfected with TLR3-specific or control siRNA were first infected with EIAV_FDDV13_. The cells were subsequently infected with EIAV_UK3_ at different times; at 72 h later, the viral RNA copy numbers of EIAV_UK3_ in the culture medium were measured with qPCR and compared with the EIAV_UK3_ RNA copy numbers in eMDM that were not initially infected with EIAV_FDDV13_. *P < 0.05, **P < 0.01.

Furthermore, to determine whether the inhibitory effects of poly I:C on EIAV replication and infection resistance induced by EIAV_FDDV13_ infection in eMDM were reduced after TRL3 knockdown, the viral copy numbers of EIAV_UK3_ in siRNA-TLR3-transfected, siRNA-C-transfected and untransfected eMDM were analyzed after stimulation with poly I:C, and the induction of infection resistance by EIAV_FDDV13_ was evaluated in siRNA-TLR3-transfected eMDM. As shown in Figure 6A, the TLR3 ligand poly I:C induced an approximately 90% reduction of the growth of EIAV_UK3_ in equine macrophages, but failed to effectively inhibit the viral replication in the cells that had been transfected with siRNA-TLR3. More importantly, TLR3 knockdown reversed the inhibition induced by EIAV_FDDV13_ to the level of 6 h of pre-infection (Figure 6B).

## Discussion

In this study, we found that in equine macrophages, EIAV_FDDV13_ infection could induce strong resistance to subsequent infection of the heterologous virulent EIAV strain EIAV_UK3_. Furthermore, we observed that some cellular factors involved in the activation of innate immunity by EIAV_FDDV13_, which occurred primarily through TLR3 activation, were important contributors to the development of infection resistance. Such host cell responses could disturb either the entrance or replication of the virus, which results in the decline of viral RNA copies in target cells. As observed in this study, the viral RNA copies of EIAV_UK3_ in the culture supernatant of macrophages preinfected with EIAV_FDDV13_ were noticeably lower than that in the un-preinfected controls.

The phenomenon that a virally infected cell becomes resistant to subsequent infection by the same or similar viruses is referred to as superinfection resistance (SIR) [20]. In this study, Figure 1A shows that at least 10 copies of viral RNA were detected per cell 3 hpi. This level of detection suggests that every target cell was infected by EIAV prior to the second infection of EIAV_UK3_. Therefore, SIR is one of the possible mechanisms that takes part in the development of resistance to subsequent viral reinfection. The primary mechanism underlying SIR induction by HIV-1 is down-regulation of the expression of the principle HIV-1 receptor CD4 on the cell surface [20,21]. In the present study, only limited up- or down-regulation on the mRNA level of EIAV receptor ELR1 was detected in eMDM infected with either EIAV_FDDV13_ or EIAV_UK3._ This observation is consistent with previous reports that the amount of membrane-bound ELR1 was not reduced by EIAV infection [8,9]. In addition, these two EIAV strains acted similarly on ELR1 expression but induced TLR3 expression differently, which implicates that ELR1 does not play an essential role in the resistance to subsequent infection inducted by EIAV_FDDV13._ In contrast to intact membrane-bound ELR1, a 2- to 3-fold difference in the expression of sELR1, the soluble form of ELR1, was detected after infection of eMDM with EIAV_FDDV13_, but not EIAV_UK3_. Because soluble viral receptors generally exert inhibitory effects on viral infection [22–24] and sELR1 mRNA accounts for as much as 50% of the total ELR1 mRNA present (unpublished data), the changes in sELR1 expression observed after viral infection in this study are likely to be involved in the resistance induced by EIAV. Our results demonstrate that poly I:C stimulated sELR1 expression by specifically activating TLR3 and that sELR1 expression was not up-regulated by EIAV_FDDV13_ infection of eMDM after the knockdown of TLR3 mRNA. These data support the hypothesis that TLR3 pathway activation mediates the up-regulated expression of the soluble ELR1 receptor after EIAV infection. However, one should be cautious in evaluating the role of sELR1 in SIR induced by EIAV_FDDV13_ because of the observed modest up-regulation of sELR1 mRNA expression and the absence of confirmation at the protein level, which was precluded by the low native expression of sELR1 in eMDM and the lack of a specific antibody that differentiates sELR1 from the prototype ELR1. Beside this, the linkage between TLR3 activation and sELR1 expression is not clear.

In addition to the up-regulation of soluble viral receptors, the enhanced expression of innate immunity-related factors is another mechanism that restrains viral replication and protects cells from subsequent infection. In the present study, we focused on TLR3 and IFNβ. Our results show EIAV_FDDV13_ strongly stimulated TLR3, whereas EIAV_UK3_ did not. TLR3 activation in macrophages has been shown to prevent HIV-1 infection [25], suggesting that the activation of the TLR3 signaling pathway might help macrophages to resist subsequent infection with similar viruses. In this study, stimulating TLR3 expression by poly I:C effectively prevented EIAV infection of the target cells, and TLR3 knockdown with siRNA largely reduced the antiviral effect of poly I:C. Although other pattern recognition receptors (PRR), such as retinoic acid-inducible gene protein I (RIG-I) and melanoma differentiation-associated protein 5 (MDA-5), belong to the RIG-I-like receptor (RLR) family and might also be involved in the innate immune response against EIAV infection, our results indicate that enhancing TLR3 expression alone effectively improved the ability of macrophages to resist to EIAV_UK3_ infection. On the contrary, our results also show that the resistance to subsequent infected EIAV_UK3_ induced by a TLR3 ligand, poly I:C, was noticeably lower than that induced by EIAV_FDDV13_ (90% vs 98% of inhibition), and the knockdown of TLR3 expression by siRNA only reversed 10-20% resistance to the subsequently infected virus. Even though the incomplete interference of TLR3 expression (about 65%) partially counts for the incomplete reverse of the EIAV_FDDV13_-induced SIR, other mechanisms may also be involved in, such as interfering viral entrance by the binding of EIAV gp90 surface protein to the membrane-bound receptor ELR1 [8,9].

Following TLR3 activation by EIAV_FDDV13_, IFNβ production in eMDM also significantly increased, which was consistent with IFNβ as a major downstream product of this PRR [26,27]. Considering the important role of IFNβ in innate anti-virus function, the significantly up-regulated IFNβ expression observed in eMDM infected with EIAV_FDDV13_ is considered a key contributor in the infection resistance induced by this virus. In addition to their role in antiviral function, TLR3 and IFNβ also play important roles in specific adaptive immune responses. In addition to their role in antiviral function, TLR3 and IFNβ also play important roles in specific adaptive immune responses. These include the promotion of T cell-dependent and -independent antibody responses in follicular B cells [28,29], the promotion of germinal center formation, the production of neutralizing antibodies [30] and the stimulation of the development of lymph node-resident T follicular helper cells [31]. These activated immunocytes are critical for the germinal center reaction and humoral immunity response [32]. Therefore, the enhanced up-regulation of TLR3 and IFNβ expression induced by EIAV_FDDV13_ likely contributes to the development of specific immunity against EIAV that is elicited in vivo through the inoculation with the attenuated strain.

Although the same amounts of initial viral titers were added, which were normalized by both TCID_50_ and viral RNA copy number, and these two viruses had similar replication kinetics and infectivity, EIAV_FDDV13_ and EIAV_UK3_ induced significantly different TLR3, sELR1 and IFNβ expression in eMDM. Our data indicate that besides the previous reported mechanism of competitive receptor binding by the viral surface protein, TLR3 pathway activation plays a vital role in the infection resistance induced by EIAV_FDDV13_ infection in macrophages in vitro. Therefore, these two viral strains should stimulate the dsRNA-recognizing TLR3 with different efficacies. Because the sequence variation in the genomes of EIAV_FDDV13_ and EIAV_UK3_ is approximately 25% [33,34], the dsRNA structures formed within the single-strained RNA viral genomes are considered different. In addition, EIAV_FDDV13_ consists of quasispecies with an average genomic diversity of approximately 3% while EIAV_UK3_ is derived from a proviral clone [6,12]. These differences may influence the binding affinities of PAMP (dsRNA) for TLR3 in these two EIAV strains, and appear to account for the differences in PRR activation and the expression of TLR3-associated cytokines. Besides the aforementioned speculation, another aspect under consideration is that the virulent EIAV_UK3_ may have one or more mechanisms to block or dampen the early innate immune response. Exploration of EIAV-specific mechanisms that are responsible for the suppression of innate immunity by virulent strains should be highly informative. Consequently, the results of this study provide insights that will facilitate a better understanding of the interaction between host cells and EIAV, as well as other lentiviruses.

Our data demonstrate that sELR1 and IFNβ are up-regulated when TLR3 is activated and those cells in which TLR3 is activated show enhanced resistance to EIAV_UK3_ infection. Silencing TLR3 expression with siRNA significantly reduces this inhibitory effect. More importantly, infection resistance induced by EIAV_FDDV13_ is significantly reversed after TLR3 silencing. Based on the significant difference in TLR3 expression in eMDM stimulated with EIAV_FDDV13_ and EIAV_UK3_, we hypothesize that TLR3 pathway activation plays an important role in the induction of infection resistance by EIAV_FDDV13_ infection in macrophages in vitro.

## Additional files

Additional file 1:
**Identification of the differentiation of macrophages from monocytes.** (A) Equine MDM were prepared from horse PBMC as described in Materials and Methods and were examined by immunofluorescence assay (IF) using a macrophage specific CD68 mAb. The adherent cells were photographed at 200× magnification. The irregular cytoplasm (grey arrow) and pseudopodia (black arrow) of phagocyte morphology were developed and observed at 48 h of cultivation under white light. Increasing signals of CD68 were detected by IF (red fluorescence). (B) The adherent cells were infected by EIAV_FDDV13_ after 48 of cultivation. Infected cells were detected by indirect IF using an EIA positive serum and a Texas Red-labeled (a red fluorescent dye) anti-horse IgG mAb 48 hpi. Additional file 2:
**mRNA detected by bDNA.** The specific oligonucleotide probe sets for the target genes included equine TLR3, TLR7, TLR8, TLR9, IFNα1, IFNβ, ELR1, and β-actin, which were used with the QuantiGene 2.0 Reagent Systems designed and provided by the manufacturer (Panomics). Additional file 3:
**Analysis of the early (Annexin V+/PI−) and late (Annexin V+/PI+) apoptotic populations of eMDM infected with either EIAV**_**FDDV13**_ **or EIAV**_**UK3.**_ eMDM infected with either EIAV_FDDV13_ or EIAV_UK3_ for 1, 3, 5 and 8 days were analyzed for apoptosis by flow cytometry. The eMDM were trypsinized and collected at the indicated time points and washed once with the Binding Buffer. These cells were re-suspended in 100 μL Binding Buffer and were stained by adding 5 μL AnnexinV and 5 μL of 3 μM PI. After 15 min incubation at room temperature, apoptotic populations were analyzed. Triplicate wells of cells were examined for each treatment. The results were calculated from the data of three independent experiments. Additional file 4:
**The expression of “house-keeping” gene β-actin in eMDM infected with EIAV or treated with Poly I:C.** Cells were infected with either EIAVF_DDV13_ or EIAV_UK3_ or treated with Poly I:C. The same amount of cells (1 × 10^5^) from each treatment was harvested and mRNA copies of β-actin in these cells were quantified by real time RT-PCR. NC: negative control of untreated cells. Additional file 5:
**Regulation of TLR7, TLR8 and TLR9 expression in eMDM by EIAV**_**FDDV13**_ **and EIAV**_**UK3.**_ Total RNA was extracted from eMDM infected with equal infectious titers of either EIAV_FDDV13_ or EIAV_UK3_ for various times. The mRNA levels of TLR7, TLR8 and TLR9 were quantified with the bDNA assay. The values of Y axis were treated by Log2. ***P* < 0.01. Additional file 6:
**Regulation of IFNα expression in eMDM by EIAV**_**FDDV13**_ **and EIAV**_**UK3.**_ Total RNA was extracted from eMDM infected with equal infectious titers of EIAV_FDDV13_ or EIAV_UK3_ for various times. mRNA encoding IFNα1 was quantified with the bDNA assay. The protein expression level of IFNα was measured using an ELISA kit. The values of Y axis were treated by Log2. **P* < 0.05, ***P* < 0.01.

## Abbreviations

EIAV: Equine infectious anemia virus
eMDM: Equine monocyte-derived macrophages
sELR1: Soluble ELR1
TLR: Toll like receptor
IFN: Interferon
TCID_50_: Median tissue culture infective dose method
PRR: Pattern recognition receptors
PAMP: Pathogen associated molecular pattern
FDD: Fetal donkey dermal
PBS: Phosphate-buffered saline
FBS: Fetal bovine serum
RT: Reverse transcriptase activity
RT-PCR: Reverse transcription PCR
bDNA: Branch DNA
ELISA: Enzyme-linked immunosorbent assays
