# Supplementary figures and images for: Infection of equine monocyte-derived macrophages with an attenuated equine infectious anemia virus (EIAV) strain induces a strong resistance to the infection by a virulent EIAV strain
Source: Vet Res. 2014 Aug 9;45(1):82. doi: 10.1186/s13567-014-0082-y (PMC4283155; doi:10.1186/s13567-014-0082-y)

## Slide 1
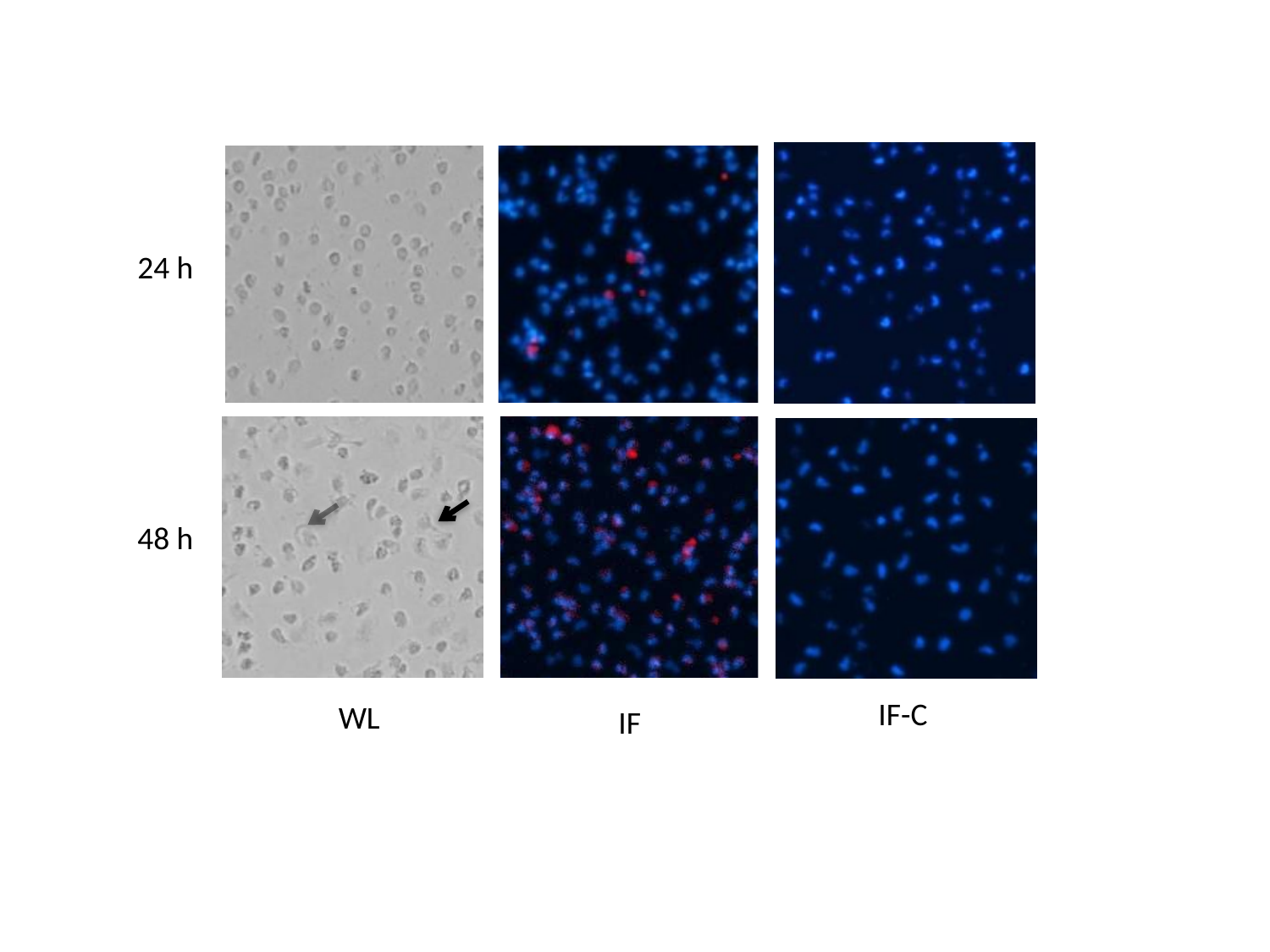

24 h
48 h
IF-C
WL
IF

## Slide 2
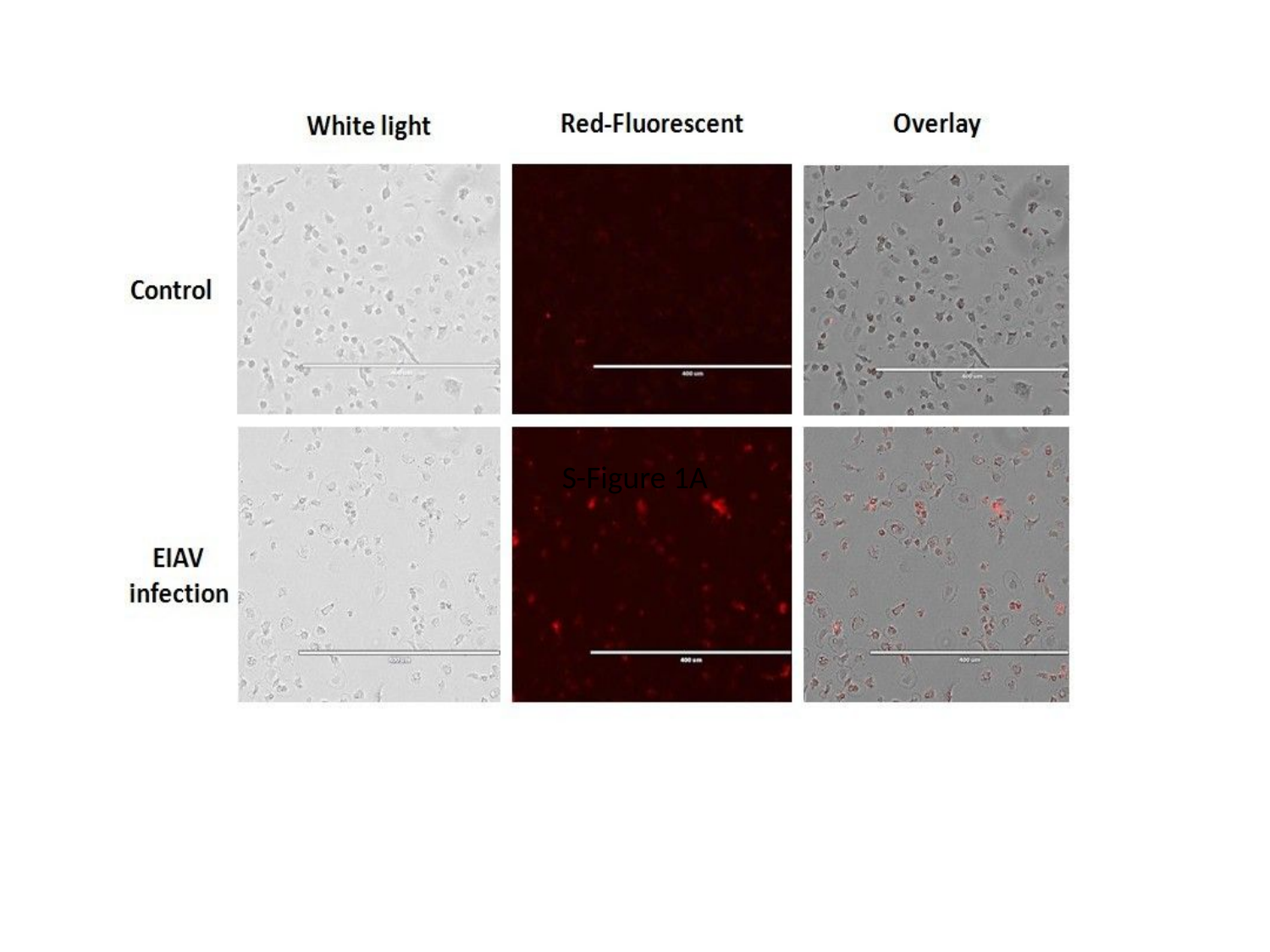

S-Figure 1A

Supplement: Additional file 1: — Identification of the differentiation of macrophages from monocytes. (A) Equine MDM were prepared from horse PBMC as described in Materials and Methods and were examined by immunofluorescence assay (IF) using a macrophage specific CD68 mAb. The adherent cells were photographed at 200× magnification. The irregular cytoplasm (grey arrow) and pseudopodia (black arrow) of phagocyte morphology were developed and observed at 48 h of cultivation under white light. Increasing signals of CD68 were detected by IF (red fluorescence). (B) The adherent cells were infected by EIAVFDDV13 after 48 of cultivation. Infected cells were detected by indirect IF using an EIA positive serum and a Texas Red-labeled (a red fluorescent dye) anti-horse IgG mAb 48 hpi. [file 13567_2014_82_MOESM1_ESM.pptx]

## Slide 1
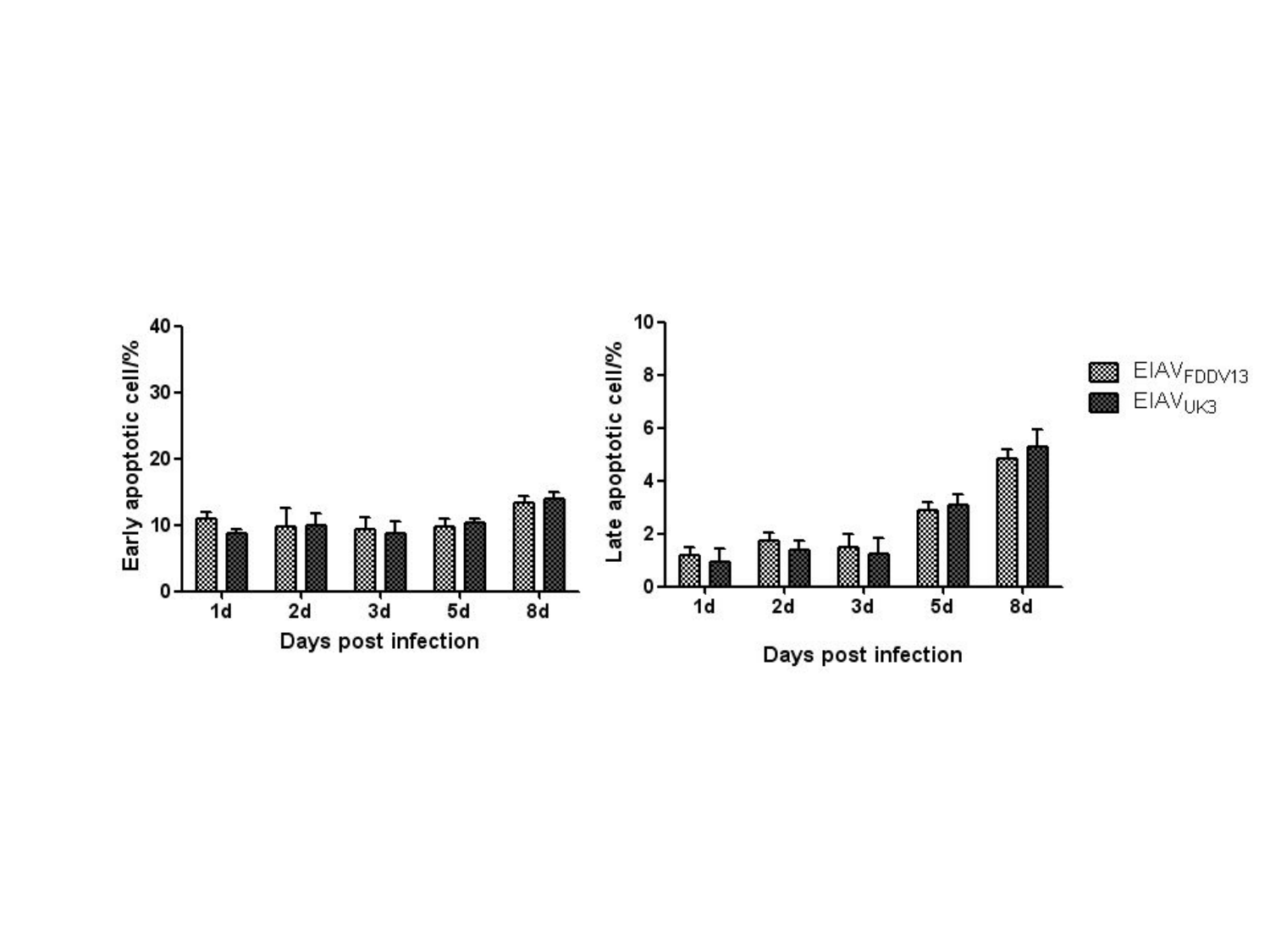

Supplement: Additional file 3: — Analysis of the early (Annexin V+/PI−) and late (Annexin V+/PI+) apoptotic populations of eMDM infected with either EIAV FDDV13 or EIAV UK3. eMDM infected with either EIAVFDDV13 or EIAVUK3 for 1, 3, 5 and 8 days were analyzed for apoptosis by flow cytometry. The eMDM were trypsinized and collected at the indicated time points and washed once with the Binding Buffer. These cells were re-suspended in 100 μL Binding Buffer and were stained by adding 5 μL AnnexinV and 5 μL of 3 μM PI. After 15 min incubation at room temperature, apoptotic populations were analyzed. Triplicate wells of cells were examined for each treatment. The results were calculated from the data of three independent experiments. [file 13567_2014_82_MOESM3_ESM.pptx]

## Slide 1
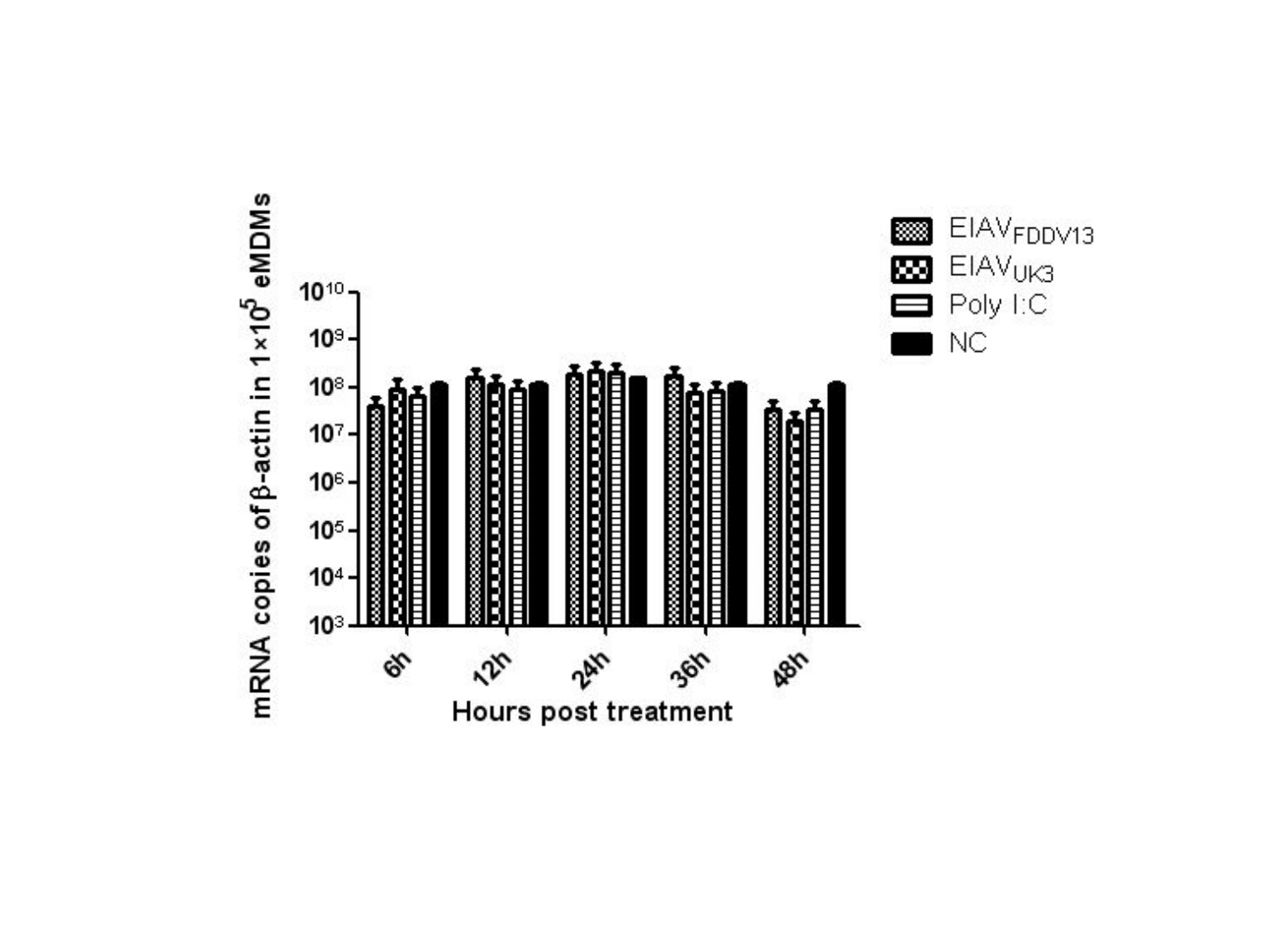

Supplement: Additional file 4: — The expression of “house-keeping” gene β-actin in eMDM infected with EIAV or treated with Poly I:C. Cells were infected with either EIAVFDDV13 or EIAVUK3 or treated with Poly I:C. The same amount of cells (1 × 10^5^) from each treatment was harvested and mRNA copies of β-actin in these cells were quantified by real time RT-PCR. NC: negative control of untreated cells. [file 13567_2014_82_MOESM4_ESM.pptx]

## Slide 1
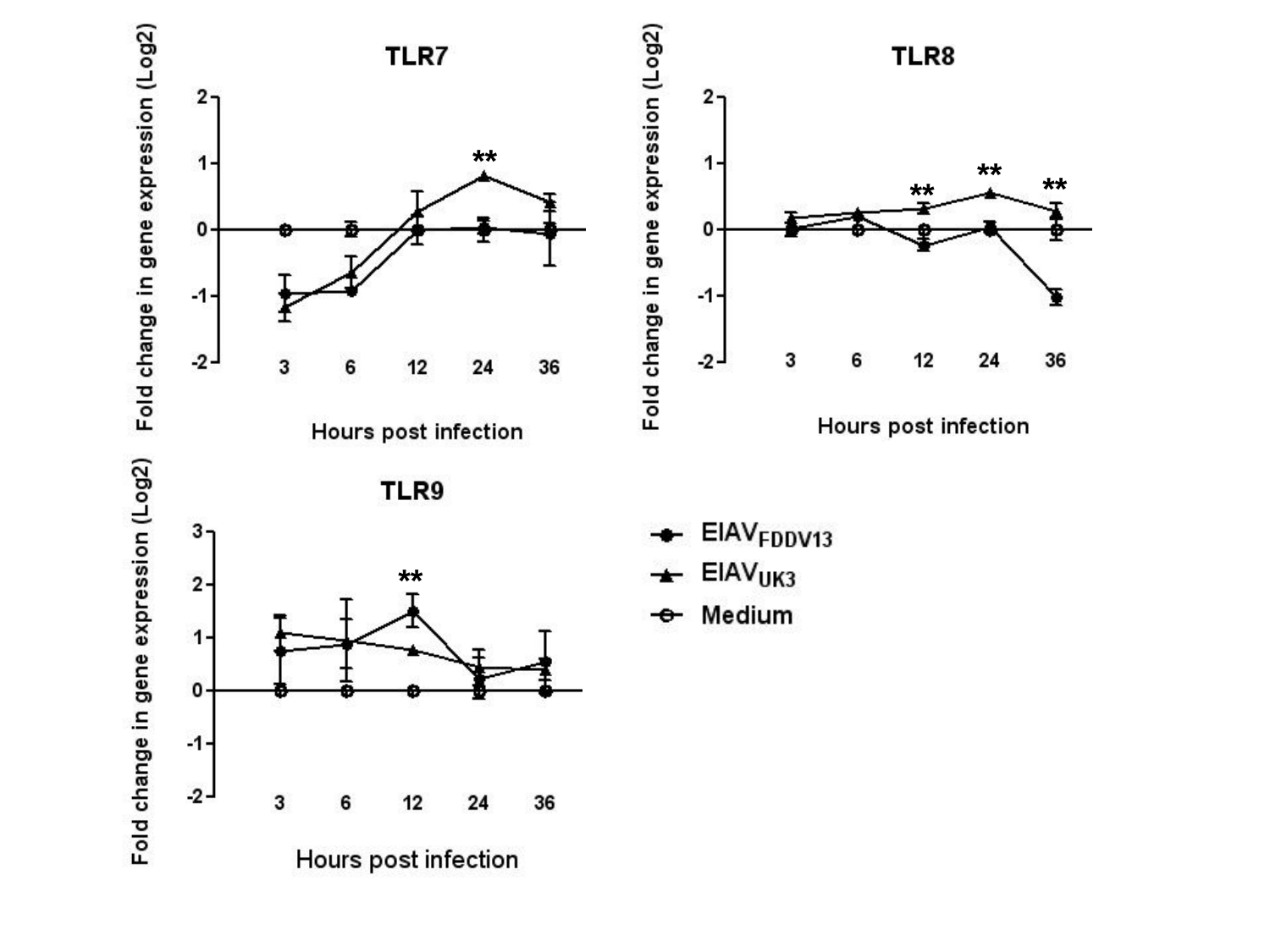

**
**
**
**
**

Supplement: Additional file 5: — Regulation of TLR7, TLR8 and TLR9 expression in eMDM by EIAV FDDV13 and EIAV UK3. Total RNA was extracted from eMDM infected with equal infectious titers of either EIAVFDDV13 or EIAVUK3 for various times. The mRNA levels of TLR7, TLR8 and TLR9 were quantified with the bDNA assay. The values of Y axis were treated by Log2. **P < 0.01. [file 13567_2014_82_MOESM5_ESM.pptx]

## Slide 1
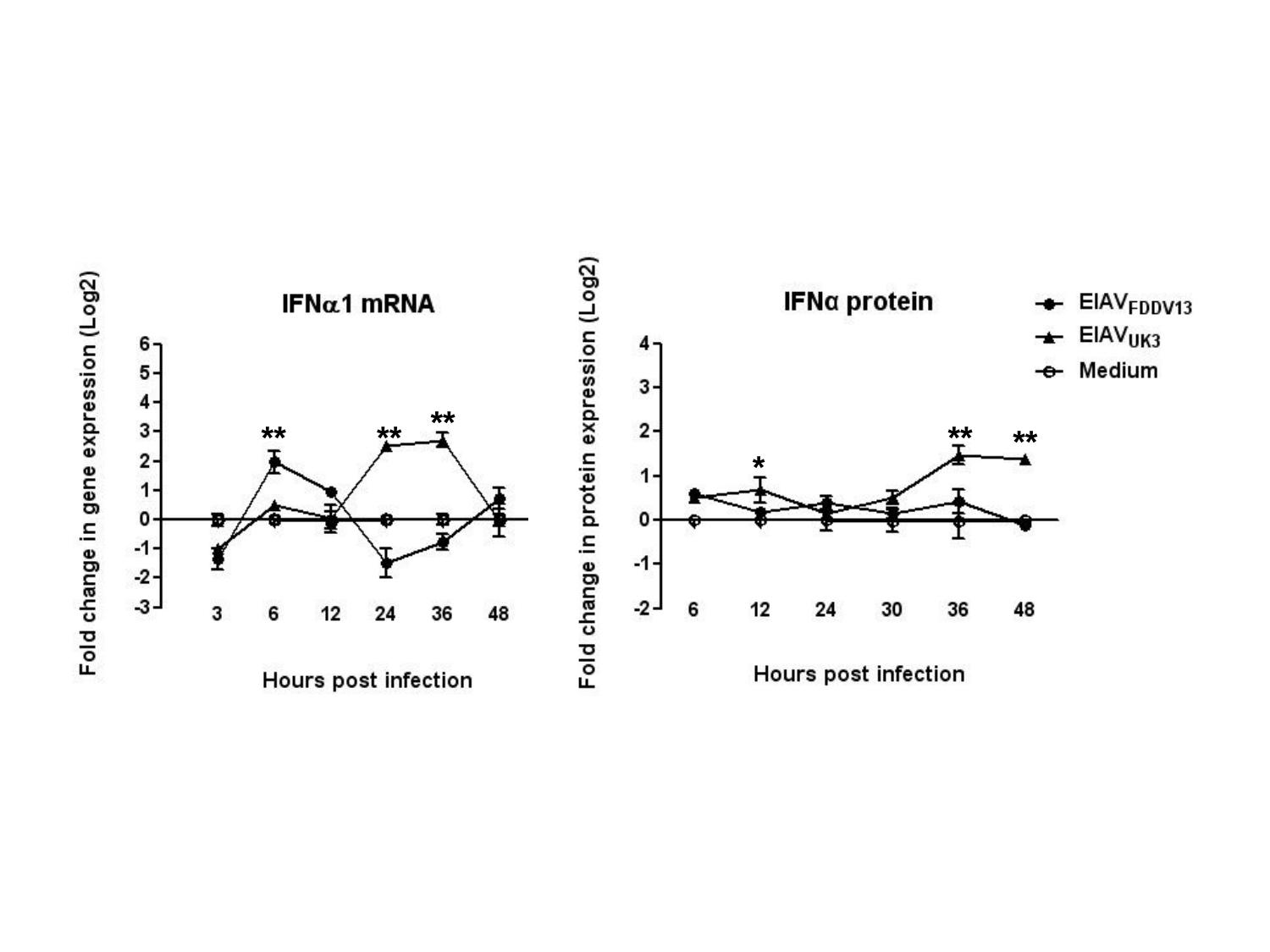

**
**
**
**
**
*

Supplement: Additional file 6: — Regulation of IFNα expression in eMDM by EIAV FDDV13 and EIAV UK3. Total RNA was extracted from eMDM infected with equal infectious titers of EIAVFDDV13 or EIAVUK3 for various times. mRNA encoding IFNα1 was quantified with the bDNA assay. The protein expression level of IFNα was measured using an ELISA kit. The values of Y axis were treated by Log2. *P < 0.05, **P < 0.01. [file 13567_2014_82_MOESM6_ESM.pptx]
