# Supplementary material for: Infection of equine monocyte-derived macrophages with an attenuated equine infectious anemia virus (EIAV) strain induces a strong resistance to the infection by a virulent EIAV strain
Source: Vet Res. 2014 Aug 9;45(1):82. doi: 10.1186/s13567-014-0082-y (PMC4283155; doi:10.1186/s13567-014-0082-y)
Supplement: Additional file 2: — mRNA detected by bDNA. The specific oligonucleotide probe sets for the target genes included equine TLR3, TLR7, TLR8, TLR9, IFNα1, IFNβ, ELR1, and β-actin, which were used with the QuantiGene 2.0 Reagent Systems designed and provided by the manufacturer (Panomics). [file 13567_2014_82_MOESM2_ESM.docx]

| Symbol | Sequence Length (nt) | Probe Set Region | GenBank Accession Number |  |
| --- | --- | --- | --- | --- |
| TLR3 | 2983 | 551-1005 | NM_001081798 | |
| TLR7 | 3749 | 1262-1730 | NM_001081771 | |
| TLR8 | 3343 | 273-736 | NM_001111301 | |
| TLR9 | 3371 | 1596-1956 | NM_001081790 | |
| IFNα1 | 555 | 2-479 | NM_001099441 | |
| IFNβ | 561 | 28-451 | NM_001099440 | |
| ELR1 | 1611 | 454-832 | NM_001081907 | |
| β-actin | 1128 | 386-706 | XM_001503888 | |
